# Training Vegetable Parenting Practices Through a Mobile Game: Iterative Qualitative Alpha Test

**DOI:** 10.2196/games.4081

**Published:** 2015-07-24

**Authors:** Leah Brand, Alicia Beltran, Richard Buday, Sheryl Hughes, Teresia O'Connor, Janice Baranowski, Hafza R Dadabhoy, Cassandra S Diep, Tom Baranowski

**Affiliations:** ^1^Children's Nutrition Research CenterDepartment of PediatricsBaylor College of MedicineHouston, TXUnited States; ^2^ArchimageHouston, TXUnited States

**Keywords:** mobile games, games for health, serious games, pediatric nutrition, parenting

## Abstract

**Background:**

Vegetable consumption protects against chronic diseases, but many young children do not eat vegetables. One quest within the mobile application Mommio was developed to train mothers of preschoolers in effective vegetable parenting practices, or ways to approach getting their child to eat and enjoy vegetables. A much earlier version of the game, then called Kiddio, was alpha tested previously, but the game has since evolved in key ways.

**Objective:**

The purpose of this research was to alpha test the first quest, substantiate earlier findings and obtain feedback on new game features to develop an effective, compelling parenting game.

**Methods:**

Mothers of preschool children (n=20) played a single quest of Mommio 2 to 4 times, immediately after which a semi-structured interview about their experience was completed. Interviews were transcribed and double coded using thematic analysis methods.

**Results:**

Mothers generally liked the game, finding it realistic and engaging. Some participants had difficulties with mechanics for moving around the 3-D environment. Tips and hints were well received, and further expansion and customization were desired.

**Conclusions:**

Earlier findings were supported, though Mommio players reported more enjoyment than Kiddio players. Continued development will include more user-friendly mechanics, customization, opportunities for environment interaction, and food parenting scenarios.

## Introduction

Vegetable consumption is protective against several chronic diseases [1,2]. Children’s dietary practices tend to track into adulthood [3], and parents play an important role in establishing healthy dietary habits in their young children [4], but often report difficulty getting their child to eat vegetables [5,6].

Because traditional interventions to increase child vegetable intake have had little or no effect [7], innovative approaches are needed. Serious games provide a behavioral intervention opportunity to increase child vegetable intake, which has had a positive impact on health-related behavior change [8]. A mobile game, *Mommio*, designed to teach parents of young children about vegetable parenting practices [9] is currently under development. Games for health predicated on a combination of social cognitive [10] and self-determination theories [11] are believed to increase likely effectiveness in promoting behavior change. According to social cognitive theory, a game that provides a player with relevant knowledge, skills, self-efficacy, and motivation is likely to result in behavior change [12]. In self-determination theory, personal values are an aspect of relatedness; fulfilling values should increase intrinsic motivation [12]. In *Mommio,* targeted desired behavior includes use of appropriate vegetable parenting practices [13]. A recent meta-analysis revealed that games were most likely to contribute to learning and behavior change when end users were involved as testers of game mechanics (A. DeSmet, written communication, January, 2015). The purpose of this alpha test was to obtain feedback from mothers of preschoolers on *Mommio* game mechanics at an early stage when changes could still be made.

A prototype version of *Mommio*, *Kiddio*, was tested by mothers who were similarly interviewed about their experience [14]. Substantial changes (eg 3D environment replacing a 2D environment, enhanced recipe selections, additional interactive items) were made. The current alpha test (ie testing game features when changes can still be made) of one *Mommio* quest was to reaffirm conclusions drawn from *Kiddio* testing [14], as well as obtain feedback on new, more sophisticated features to produce an effective game that trains vegetable parenting practices among mothers of young (3-5 year old) children.

## Methods

### Sample and Recruitment

Mothers of 3-5 year old children were recruited after obtaining approval from the Baylor College of Medicine’s Institutional Review Board. Mothers who reported no difficulty getting their child to eat vegetables or were not 20-40 years old were excluded. Recruiting took place through digital and printed flyers distributed throughout the Texas Medical Center, and from the Children’s Nutrition Research Center’s volunteer list. All mothers (n=20, demographics described in Table 1) provided informed consent.

### Game Description

*Mommio* is a first-person role playing video game that simulates a mother interacting with her preschool age child (called “Kiddio”). Kiddio hates veggies. Players can customize their Kiddio’s name, gender, skin, and hair color. The game takes place in a 3D world that includes the player’s virtual house, complete with working kitchen and numerous common distractions, such as a begging family dog and booming televisions. The player navigates by double-tapping the ground to move inside the world and using an onscreen thumb stick to change perspective. Single tapping a character (Kiddio or the dog) or a game object (refrigerator, food item, etc.) starts an interaction. At mealtime, Kiddio prompts the player to action by exclaiming “I’m hungry.” The player must then select a vegetable recipe from the kitchen’s recipe box as a side dish for lunch. The recipe box features simple recipes for a variety of vegetables, and non-veggie recipes (eg macaroni and cheese) which result in a loss if selected. Once the player and Kiddio are seated at the kitchen table, the player tries to get Kiddio to eat the vegetable, and Kiddio refuses.

*Mommio* is not an easy game to win. The player can try different food parenting strategies, such as choosing from effective and ineffective statements to say to Kiddio, or modifying the environment (eg turning off the television). The variety of statements that can be selected to say to Kiddio could be modified by voice tone (gentle, firm, or harsh) and facial expressions (happy, neutral, concerned, or angry). If the player makes an effective move, she comes closer to winning the game. If she selects ineffective ways for dealing with the child, she moves toward losing the game. At the conclusion of each *Mommio* quest, Kiddio either tastes the vegetable to signify a victory, or runs out of the room, indicating a loss.

At the conclusion of the quest, players are led through a series of screens that asks them to select a parenting value that is important to them, from which tailored motivational messages will be delivered, as well as a plan for strategies to use at home to increase vegetable consumption based on the selected value.

**Table 1:** Demographics.

Demographics		n (%)
**Child Gender**		
	Boy	13 (65%)
	Girl	7 (35%)
**Highest Education Completed**		
	High School graduate or GED	1 (5%)
	Technical school	2 (10%)
	Some college	7 (35%)
	College graduate	5 (25%)
	Post Graduate Study	5 (25%)
**Annual Household Income**		
	Less than $30,000	4 (20%)
	$30,000 to $60,000	8 (40%)
	Over $60,000	8 (40%)
**Ethnicity**		
	Hispanic	9 (45%)
	African American	6 (30%)
	White	3 (15%)
	Asian-Non Vietnamese	1 (5%)
	American Indian	1 (5%)
**Employed**		
	Yes	14 (70%)
	No	6 (30%)
**Primary Responsibility for** **Feeding the Selected Child**		
	Me	13 (65%)
	Shared among multiple people	7 (35%)
**Marital Status**		
	Married or living with a significant other	13 (65%)
	Single, Never married	6 (30%)
	Divorced. Separated, or Widowed	1 (5%)

### Procedures

Mothers scheduled one hour time slots at their convenience to play *Mommio* on an Apple iPad tablet under researcher observation. Qualitative interviews about their experience followed, conducted by a trained interviewer. Participants were provided a player tutorial guide upon starting their game session, and were encouraged to ask questions. The interview contained 27 questions, with additional probes and prompts (Textbox 1). At the conclusion of the interview, participants were thanked and compensated $25.

**Interview questions.**

1. What do you think the game is about?
2. What, if anything, did you like about playing the game?
3. What, if anything, did you not like about playing the game?
4. What do you think about the name of the game?
5. What did you think about personalizing the child character?
6. What did you think about being able to pick different things to say to the child character?
7. What did you think about the child character’s reactions to your statements?
8. What did you think about being able to choose recipes to offer the child character?
9. What things did you do in the in the game that you could use with your own child?
10. What did you think about the artwork or graphics for the game?
11. What did you think about moving around the house?
12. What did you think about interacting with items in the game environment?
13. How do you think you can win this game?
14. What did you think about the possibility of game points?
15. What do you think about receiving feedback at the end of gameplay?
16. What did you think about the game’s question about “What is most important to you”?
17. What did you think about the “most important to you” choices offered?
18. What are your thoughts on receiving tips to practice at home?
19. What did you think about having a website as an additional resource?
20. Today you played the “lunch” level of the game. The finished game will include other situations like car trips, dinners, grocery stores, and fast food restaurants. Would you play a game like this?
21. Do you read nutrition labels while at home or in the grocery store?
22. If the game was a free app game how likely would you download it to play?
23. How much would you be willing to pay for this app game?
24. If you had to pay for this app game, how likely would you download it to play?
25. On a scale of 1 to 3, where 1 is “not difficult” and 3 is “very difficult,” how difficult is it to get your child to eat vegetables?
26. Using a 1 to 4 STAR rating scale, how would you rate the game?
27. Before we finish, is there anything else you want to tell me that you haven’t had an opportunity to say?

### Data Analysis

Interviews were audio-recorded and transcribed in full. All transcriptions were double-checked against the original recording before importing into NVivo (Version 10.0, 2012, Doncaster, VIC, Australia). Thematic analysis [15] was used to code each response within the questions posed. Transcripts were double coded to ensure reliability. Differences between coders were adjudicated by discussion and consensus.

## Results

### Game Elements

Most mothers completed three iterations of the game. On average, gameplay consumed 20 (SD 7) minutes. Patterns emerged in regard to the design of the *Mommio* game. These themes reflected on game look and feel, as well as mechanics not related to vegetable parenting.

**Figure 1:**
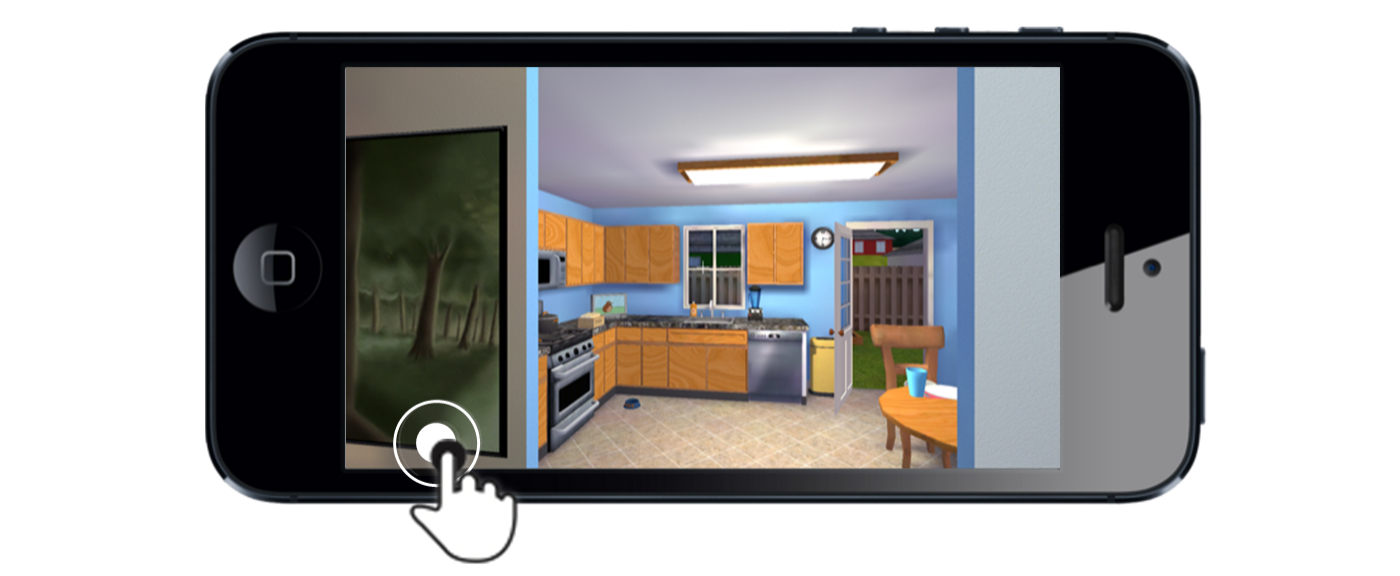
Joystick mechanic.

A majority of the sample (12/20, 60%) found navigating the game environment frustrating. This was largely due to the thumb stick, which was felt to be *“overly sensitive”* and *“very hard to control”* (see Figure 1). Over half of the mothers (12/20, 60%) found the circle thumb stick difficult to use, and a few (2/20, 10%) found the feature to be a deterrent to engagement. For some (6/20, 30%), the thumb stick mechanic was a learning curve that *“took me a while to get accustomed to.”* A handful of mothers (3/20, 15%) suggested using direction arrows instead of or in conjunction with the thumb stick, or to maneuver by dragging *“not the circle, just with your finger.”* Less than a quarter of mothers (4/20, 20%) mentioned liking the thumb stick feature.

Nearly all mothers (19/20, 95%) enjoyed the ability to personalize the look of the child character, although some (5/20, 25%) wanted more hair options while others (7/20, 35%) wanted more clothing options, which *“would feel more like it’s my child.”* Nearly all participants (19/20, 95%) enjoyed naming the character after their own child, which increased the realism of the story. A majority of participants (16/20, 80%) would have liked to pick the child’s personality (ie temperament [16]) in the game, as this would be

more realistic to what parents or guardians have to deal with when they’re trying to encourage a kid with a certain type of personality to eat their vegetables, because all kids are not the same.

The main action of the game took place in the kitchen of the home. Perhaps due to this, nearly half of the mothers (8/20, 40%) were not aware that the navigable 3D environment included the entire house and yard. This was reflected in comments such as

I wish we would have been able to move more around the house to make it a little interesting

and

I didn't try to go out past the kitchen. But I'm sure that's the only place you'll be able to go

Most (16/20, 80%) thought that double tapping the floor to move in the environment was either good or fine. Four mothers thought this countered some of the difficulty experienced with the thumb stick feature, saying “I thought that made it easier, a lot easier”*.*

However, a few (3/20, 15%) mothers did not understand or use this feature.

Thirteen mothers liked interacting with items in the game environment, with 25% (5/20) mentioning that this feature made the game more realistic. However, three mothers were

unclear whether doing any of those things has any impact on the game, like turning off the TV. Is it important to do that as part of the process or are these just things that are there for no particular reason?

All participants opened and closed cupboards in the kitchen environment, and 35% (7/20) enjoyed seeing the contents of the kitchen and nutrition labels of food. Almost half of all participants (9/20, 45%) wanted more available interactions with items in the kitchen, saying things such as

I did try to get a box of something off one of the cabinets, but that didn’t work either. So just having the option would be good.

Many participants liked interacting with the environment in other ways, such as turning off the television (9/20, 45%) and speaking to the dog (5/20, 25%), which increased the realism of the environments and was “something extra to do…it was neat”.

Three quarters of participants said that they liked the graphics and looks of the game. About one third of all parents thought that the home and kitchen looked modern or realistic (6/20, 30%), liked the bright colors used (5/20, 25%), and thought that the art looked professional (5/20, 25%); though a minority mentioned (2/20, 10%) that the art looked like cartoons tailored for preschoolers. A few participants (3/20, 15%) thought the art was passable, but unremarkable, such that they were

not sure it would win awards necessarily, in terms of its, like, graphic ability, but it was totally fine.

The kitchen and home environment were noted as “nice and cute” and about a quarter of participants (4/20, 20%) mentioned enjoying exploring and interacting with the realistic kitchen, which was stocked with grocery items.

All mothers agreed receiving feedback at the end of gameplay would be beneficial. The majority (13/20, 65%) thought that feedback should occur at the end of each episode (level), although a few (3/20, 15%) thought that it should occur after a few episodes taking place in one sitting. The most commonly preferred method of delivery was in-app, followed by email. Common requests for feedback content included points earned, quality of food choices made, tips to improve gameplay, and evaluations of parenting statements selected. Some participants (7/20, 35%) wanted to see a tie-in to the learning goals of the game through feedback*,*

because if I’m going to take time to play the game, then I would want to receive something from it beneficial that I could use.

### Learning Content

For the video game to be effective, it must adequately express and incorporate vegetable parenting messages. Several themes were found from participant discussion of learning content.

**Figure 2:**
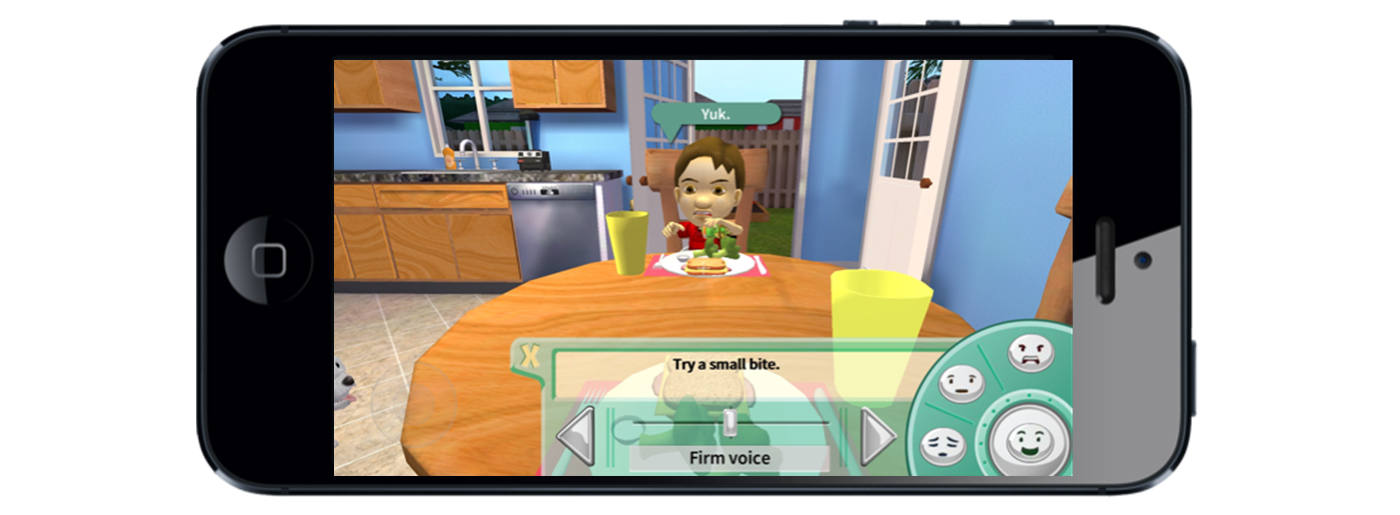
Playing a meal.

All mothers enjoyed being able to select statements to say to Kiddio and found the available statements realistic (see Figure 2). Several (6/20, 30%) mentioned the variety of statements available to them, which

was great because there were some things I wouldn't necessarily say to my child. But I found something that fit me and my personality on how I would respond in the situation.

Others (4/20, 20%) thought the selectable statements were limited and should be expanded to not get repetitive in future levels.

Every mother interviewed found statements that she would use or has used with her own child. Most commonly mentioned statement types noted as effective in the game were encouragement to try a vegetable or take a few bites and trying or preparing vegetables with the child. The most frequently mentioned ineffective strategies were bribing with food or activities after eating and telling the child that he should sit at the table until he eats his vegetables.

Many players thought that the child character’s reactions were realistic (17/20, 85%) and similar to their own child (15/20, 75%). A few mothers mentioned liking the character’s realistic attachment to a favorite toy (3/20, 15%), as well as Kiddio’s responses to player actions. Many (8/20, 40%) pointed out differences between the child character and their own child, including differences in stubbornness (3/20, 15%) (the child character was perceived as both more and less stubborn than a parent’s own child), responses to bribery (2/20, 10%) (the character does not respond well to bribery, real child does), and activity level (4/20, 20%) (own child talks and fidgets more, including eating items on plate to avoid vegetables).

While a large majority of mothers (17/20, 85%) thought Kiddio responded mostly as expected, three participants found that there was not enough variation of child animations to portray appropriate reactions, as

it seemed as if it was always stuck on a certain, ‘I don’t want it, I don’t want to do it’ kind of look.

All but one mother found the child’s reactions helpful in knowing how well they were doing in the game, as “you can read by the facial expressions” The mother who did not find the child character’s reactions helpful cited a lack of variation in expression, similar to the reasons for the child character’s unexpected reactions.

Upon first playing, game goals and objectives were unclear to many participants. Some (8/20, 40%) “had no clue what to do,” which resulted in confusion or frustration. Mothers who expressed this either consulted the provided user guide or asked the researcher for help before making strides in game progression.

Most mothers (19/20, 95%) liked the vegetable recipes provided through the in-game recipe box and that recipes included instructions and nutrition information. Some (8/20, 40%) pointed out real-world benefits for including this information, and that the recipes may

help me to learn like what's healthy, what's not healthy. I guess it's going to be good for kids as well.

The vast majority of mothers (18/20, 90%) said they would be interested in using the recipes at home with their own child, or already do. Nearly all mothers (18/20, 90%) said they would be interested in learning how to make the recipes at home. A minority of mothers (2/20, 10%) found the recipes too simple and thus already knew how to make them.

Almost half of mothers (9/20, 45%) would prefer to receive game recipes though an in-game recipe box with an option to select individual recipes to send to their email. Other preferences included only the in-game recipe box, a combination of email and website, featured in the app but outside of gameplay, and on a website. Frequent responses to ideal recipe content, aside from ingredients, included calorie count, instructions, nutrition content, and serving size.

Nearly half of all participants (9/20, 45%) thought the player wins the game by getting Kiddio to eat vegetables. Several additional mothers focused on the parenting strategies as ways to win, such as watching one’s tone while speaking (6/20, 30%), selecting the right parenting statement (4/20, 20%), and using generally effective communication strategies (2/20, 10%). Three mothers did not mention parenting strategies, but indicated that picking healthy foods was the way to win.

### Real World Application

A series of questions asked participants to reflect on personal values regarding parenting practices. This included a game prompt, which asked participants to practice vegetable parenting based on a parent-selected value. Participants generally were open to a real-life crossover, given their current values and habits.

A game storyboard featured a question asking players to select what value (e.g. being a role model, being spiritual) was most important to them given multiple options. Half of all mothers interviewed (n=10) thought this question was good or okay. However, six mothers found it confusing, commenting “I didn’t relate this to the game really,” and “I wasn't really sure of exactly what the point was there”. The vast majority (17/20, 85%) understood the question, although two mentioned they needed more context to understand what the question was asking.

A quarter of participants (5/20, 25%) found selectable value options to be good or okay. A handful of mothers (4/20, 20%) agreed that

being spiritual-even though it may be important to you, but I think it’s just so far and apart from the game

and would have liked to see this value replaced with something that fits better with the game and other available options. Most (15/20, 75%) said the selectable options made sense to them, but a couple said they were not sure how to answer given provided choices. All mothers interviewed thought at least one of the listed values applied to them, though five would have liked to select more than one value.

All mothers indicated they would be willing to try out game tips with their own child, largely willing to consider parenting practices that could help with their parenting, saying “if you can get advice and help from others, it's always a plus”. All but one mother agreed that receiving tips to practice at home would be helpful, as

it helps that you have those tips and that you've practiced, and you have some information under your belt and ready.

The mother who disagreed thought that a game was not the best format for receiving parenting tips. Preferred methods of parenting tip delivery and scheduling time to practice were email, electronic calendar app or other mobile scheduling program, in-game only, and text message.

All participants indicated at least one food parenting practice from the game they either had used in the past with their own child or would use in the future. Popular responses include being aware of vocal tonality or facial expressions when speaking to the child (see Figure 2), staying positive or encouraging, sitting down at the table with the child for meals, role modeling healthy habits, choosing healthy vegetable recipes, involving one’s child in the meal preparation process, and general communication strategies.

Fourteen mothers reported reading nutrition labels in the grocery store. Those who read labels used the information because they wanted to feed their child healthy foods, prevent disease related to unhealthy eating, be aware of what is in their food, make choices between similar products, and limit certain nutrition elements, such as sugar.

When reading nutrition labels, mothers said they looked for numbers for sugar or carbohydrates, calories, sodium, fat, protein, and portion size. Five mothers reported looking at ingredient lists in addition to nutrition panels. Several of these components were listed on nutrition labels provided for each recipe in the game. About half of mothers interviewed (11/20, 55%) thought all recipes in the game should have these nutrition panels, and even more (14/20, 70%) wanted to see the ingredients of a recipe in the recipe box before making a selection.

### Future Production

Evaluations of the game as a whole and thoughts about its future were presented over the course of each interview. More than half of mothers interviewed (13/20, 65%) thought that a companion website would be good or helpful. Mothers suggested the website include recipes with nutrition tips, parenting tips, forums or user generated content, links to external resources, and the game itself.

A larger, more complex game that featured several mealtime contexts was of interest to a large majority of mothers (17/20, 85%). Those interested in playing said that variety would make the game more interesting and would be helpful delivering parenting tips for different scenarios. A few mothers (3/20, 15%) mentioned wanting grocery store help specifically, while a few others (2/20, 10%) mentioned tips for car trip food parenting.

All but one mother said a game that varied in context would seem more like their real life. One mother said that it would not, due to the fact that nearly all meals her family consumed were in the home.

Most mothers (15/20, 75%) said they would likely play multiple meals in a row in the proposed expanded version of the game. Those who mentioned not wanting to do this commented that “trying to remember too much at the same time is not going to be effective, either,” and that playing the game in small chunks would let them retain tips and information for future use.

When asked how likely they would be to download the game if it were free, the large majority of mothers (17/20, 85%) said that they would very likely do so. Reasons included it “would be beneficial” or useful to parenting, that the game supports interactive time with one’s own child if they play together, and curiosity. Two mothers who would not download the game for free reported not liking any types of games, and the remaining mother was not sure whether or not she would download the game until it was expanded further.

Two mothers reported being slightly less likely to download the game if they had to pay for it than if it was free. Three would not pay for the game, with one mother citing that she’d rather “research for free versus paying for something that I feel I....can gain for free”.

When asked for a rating of “hated it, didn’t like it, liked it, or loved it” most mothers (13/20, 65%) said they liked the game. Three mothers did not like the game. Four mothers said that they loved it, “because it was educational and it was fun. And it kept my attention”.

Mothers who did not say that they “loved” the game were asked what could be changed to make them love it. Suggestions included expanding the game across contexts and functionalities (as discussed in the question about game expansion). Mothers also requested clarification of game goals/objective, perhaps through the use of a tutorial, as well as improvement to game navigation by modifying the thumb stick feature. More things to do in the kitchen, such as picking up pantry items or cooking were requested. Mothers also felt additional recipes and feedback after gameplay would make the game better, as well as the addition of audio.

## Discussion

Results were generally consistent with earlier findings [14]. Both studies uncovered initial confusion by players about the game’s objective, but understanding of the game’s primary goal after at least one level was played [14]. Mechanics and navigation were considered difficult in both studies [14], although they had been modified after the earlier test. Customization was appealing for both the child character as was the ability to choose what to say and do in the game environment [14]. Feedback was a desirable in-app feature across both studies, as was Kiddio’s animations and reactions, though both samples suggested ways they could be improved [14]. Both studies found that while asking players to choose a value was fine, mothers had a difficult time selecting only one parenting value given the options displayed, though these options were different in form and number for each study [14].

Some findings between *Kiddio* and *Mommio* differed [14], perhaps due to enhancements in the game itself. *Mommio* participants rated the game higher; with more saying they “loved” the game [14]. Many more *Mommio* than *Kiddio* players were interested in playing the game if it were free or if it had a small cost, suggesting the changes made enhanced its appeal.

Aspects of social cognitive and self-determination theories were endorsed. Participants reported gaining knowledge through situated learning and environmental exploration; transferable real-world skills were learned through parenting statement selection. Self-efficacy was experienced through personal success in winning the game and expressed by vocalized interest in trying new methods at home. Intrinsic motivation for selecting effective vegetable parenting practices was enhanced through motivational messages tailored to parent-selected value statements. All these findings support the likelihood that *Mommio* will influence behavior change.

Repeated testing of an evolving serious game is valuable. Many earlier findings were supported, strengthening their original import. Differences between the studies demonstrated that game has evolved in an effective way. Several changes will be made to address issues raised (Textbox 2). Trouble with mechanics will be resolved through the discarding of the thumb stick in favor of finger-sweep controls. A tutorial level and more overt Kiddio expressions will be added to address confusion with these game elements.

**Directives for future changes to Kiddio.**

- Change or delete onscreen circle thumb stick feature
- Expand game across additional levels and contexts
- Expand recipes
  - Add more recipes
  - Add “send to email” feature
  - Keep nutrition panel with carbs, fat, sodium, protein, calories and portion size
- Add a tutorial level
- Add feedback, occurring at the end of each game, in the app itself, that includes parenting tips
- Add more interaction to kitchen items, such as ability to select food from the cabinets
- Add a broader range of Kiddio facial expressions, especially for mealtime interactions
- Add more customization of the child character’s physical appearance (clothes, hair)
- Keep tone/expression mechanic and selectable statement variety
- Keep value statement, but add a sentence for more context
  - Either replace “spiritual” answer or add many more selectable values that add balance

Increased customization of player experience was desired. This will be addressed by adding more options to modify Kiddio, such as hairstyles, and more ways to interact with the kitchen. *Mommio’s* new features, such as inclusion of recipes and more environment interactions, were well received. Thus, recipes will be increased in number and detail, and made available for home use. Mothers liked the idea of expanding the game, and reported expansion would increase their desire to play. The final *Mommio* game will expand across environments such as the car and grocery store.

Both samples wanted feedback and tips, thus feedback will become more detailed in future versions of the game. Current positively evaluated features included selectable statements, voice tone selection, and value statements, which will be kept in future versions of *Mommio.* However, the mechanic for selecting values may evolve.

This study’s limitations include a small sample, and only one game quest of one episode of the game was tested. The game was presented on a tablet, which was familiar to some, but not to others, and may have served as a barrier to engagement.
